# Development and validation of the Newcastle laryngeal hypersensitivity questionnaire

**DOI:** 10.1186/1745-9974-10-1

**Published:** 2014-02-19

**Authors:** Anne E Vertigan, Sarah L Bone, Peter G Gibson

**Affiliations:** 1 Speech Pathology Department, John Hunter Hospital, Locked Bag 1, Hunter Region Mail Centre, Newcastle, NSW 2310, Australia; 2 Hunter Medical Research Institute, Newcastle, Australia; 3 University of Newcastle, Newcastle, Australia; 4 Department of Respiratory and Sleep Medicine, John Hunter Hospital, Newcastle, Australia

**Keywords:** Laryngeal hypersensitivity, Chronic refractory cough, Vocal cord dysfunction, pdoxical vocal fold movement, Globus pharyngeus, Muscle tension dysphonia, Laryngeal hypersensitivity syndrome

## Abstract

**Background:**

Laryngeal hypersensitivity may be an important component of the common disorders of laryngeal motor dysfunction including chronic refractory cough, pdoxical vocal fold movement (vocal cord dysfunction), muscle tension dysphonia, and globus pharyngeus. Patients with these conditions frequently report sensory disturbances, and an emerging concept of the ‘irritable larynx’ suggests common features of a sensory neuropathic dysfunction as a part of these disorders. The aim of this study was to develop a Laryngeal Hypersensitivity Questionnaire for patients with laryngeal dysfunction syndromes in order to measure the laryngeal sensory disturbance occurring in these conditions.

**Methods:**

The 97 participants included 82 patients referred to speech pathology for behavioural management of laryngeal dysfunction and 15 healthy controls. The participants completed a 21 item self administered questionnaire regarding symptoms of abnormal laryngeal sensation. Factor analysis was conducted to examine correlations between items. Discriminant analysis and responsiveness to change were evaluated.

**Results:**

The final questionnaire comprised 14 items across three domains: obstruction, pain/thermal, and irritation. The questionnaire demonstrated significant discriminant validity with a mean difference between the patients with laryngeal disorders and healthy controls of 5.5. The clinical groups with laryngeal hypersensitivity had similar abnormal scores. Furthermore the Newcastle Laryngeal Hypersensitivity Questionnaire (LHQ) showed improvement following behavioural speech pathology intervention with a mean reduction in LHQ score of 2.3.

**Conclusion:**

The Newcastle Laryngeal Hypersensitivity Questionnaire is a simple, non-invasive tool to measure laryngeal pesthesia in patients with laryngeal conditions such as chronic cough, pdoxical vocal fold movement (vocal cord dysfunction), muscle tension dysphonia, and globus pharyngeus. It can successfully differentiate patients from healthy controls and measure change following intervention. It is a promising tool for use in clinical research and practice.

## 

Laryngeal Dysfunction Syndromes include chronic refractory cough, pdoxical vocal fold movement (vocal cord dysfunction), muscle tension dysphonia and globus pharyngeus. These conditions present to clinicians as discrete syndromes based around a dominant manifestation of a disordered laryngeal adductor reflex, e.g. cough, vocal fold closure, vocalisation, or swallowing [1,2]. They are not usually associated with structural change in the larynx, and patients with these conditions are frequently referred to speech pathology for behavioural management. An emerging concept is that of the Laryngeal Hypersensitivity Syndrome, where a sensory hyperresponsiveness is observed to be a relevant component of the laryngeal dysfunction syndromes [1,3-10]. Laryngeal discomfort, which in some circumstances suggests the presence of a sensory neuropathic disorder [7,8], is present in these conditions however there is a lack of validated and easily administered instruments to assess this discomfort.

Abnormal sensory experience is characterised in three ways: (1) hypersensory–sensation triggered by stimuli that is sub-threshold for triggering that sensation, (2) pesthesia–altered sensory experience, and (3) allodynia–sensation triggered by stimuli that do not normally trigger those sensations. This characterisation can be applied to the concept of abnormal laryngeal sensation [7,8].

Patients with laryngeal dysfunction syndromes such as chronic refractory cough, pdoxical vocal fold movement, globus pharyngeus and muscle tension dysphonia frequently report irritation and discomfort in the laryngeal region. The focus of treatment in these conditions involves motor rather than sensory areas of dysfunction. There are standardised tests to measure sensory laryngeal dysfunction such as cough reflex sensitivity testing, Fibreoptic Endoscopic Evaluation with Sensory Testing (FEEST) and hypertonic saline challenge. While these tests provide objective reliable data they are expensive to administer and are rarely available outside of specialist treatment areas. Furthermore, these tests do not quantify the patient experience of the discomfort. Several questionnaires exist to measure quality of life in patients with voice disorders [11] and chronic cough [12]. However there are no standardised questionnaires to measure the laryngeal sensation in these conditions.

In this study, we have developed and tested a questionnaire to document the laryngeal sensory abnormalities reported in these syndromes. The aim of this study was to develop a Laryngeal Hypersensitivity Questionnaire for patients with laryngeal dysfunction syndromes and to measure the laryngeal sensation occurring in these conditions. This paper describes the development and validation of the Newcastle Laryngeal Hypersensitivity Questionnaire which is a self-rated measure of laryngeal sensation.

## Method

### Participants

A total of 97 participants were studied. For the item validation and discriminant analysis, we studied 53 participants comprising healthy controls (n = 15) and 4 clinical groups: chronic refractory cough (n = 11), pdoxical vocal fold movement (PVFM; n = 18), globus pharyngeus (n = 6), and muscle tension dysphonia(n = 3). The case groups were recruited from consecutive referrals to the speech pathology department for assessment and treatment of their laryngeal condition. Exclusion criteria for all groups included recent (past month) upper respiratory tract infection, current smoking, untreated asthma, rhinitis or gastroesophageal reflux, significant psychological factors or neurological impairment preventing participation.

For the study of questionnaire responsiveness, the questionnaire was administered to an additional group of 44 participants with laryngeal dysfunction syndromes. These participants included chronic refractory cough (n = 38), PVFM (n = 4) and globus pharyngeus (n = 2).

1. Chronic Refractory Cough. The participants with chronic refractory cough had been referred by respiratory physicians for behavioural management of cough [13]. The cough had persisted for longer than eight weeks and was refractory to medical treatment based on the anatomic diagnostic protocol and including asthma, gastroesophageal reflux disease, lung pathology and rhinosinusitis [14]. The cough was of significant concern for the patient to seek medical treatment.

2. pdoxical Vocal Fold Movement. The participants with pdoxical vocal fold movement were diagnosed by either respiratory physicians or otolaryngologists. These patients had been referred for behavioural management of their respiratory symptoms which included inspiratory dyspnoea, noisy breathing and throat tightness. Asthma and other pulmonary diseases had been discounted as a reason for the respiratory problems in this group. They had positive symptoms of PVFM and a fall in FIF50 of greater than 20%.

3. Globus Pharyngeus. The third group with globuspharyngeus were referred for clinical assessment and management of swallowing. These participants presented with globus sensation such as a sensation of an irritation, lump or tightness in the throat in the absence of oropharyngeal dysphagia.

4. Muscle Tension Dysphonia. The fourth group included patients with muscle tension dysphonia diagnosed by otolaryngologists referred for dysphonia. These patients had a deviation in perceptual voice quality along with excessive tension in the intrinsic and/or extrinsic laryngeal muscles [15] in the absence of any structural, neurological or significant psychological pathology.

5. Healthy Controls. The healthy controls were recruited from the Hunter Medical Research Institute Healthy Control Register (n = 10), from a previous study where they served as healthy controls (n = 2) and by word of mouth (n = 3). All healthy controls had no history of voice disorder, chronic cough or extrathoracic airway hyperrresponsiveness. Voice was judged as within normal limits by a qualified speech pathologist. Healthy controls were also excluded if there was presence of asthma, presence of post-nasal drip syndrome, presence of gastro-oesophageal reflux, symptoms of breathing, cough or voice difficulty and/or swallowing difficulty.

Written consent was obtained for all participants. The study was approved by the Hunter New England Research Ethics Committee.

### Procedure

Item generation: Potential questionnaire items were generated by reviewing literature regarding sensations in chronic pain and neuropathy and from previous patient reports of laryngeal discomfort [8]. These reports had been generated from semi-structured interviews involving open ended questions between patients and clinical staff. Questions in these interviews included “How does your throat feel?” and “Can you describe the sensation in your throat?”. Specifically, we sought examples of hypersensory experience, altered sensory experience or sensations triggered by stimuli that do not usually trigger these sensations. This latter example is a sensory experience corresponding to allodynia, and paroxysms are triggered by sensory exposure. These items were compiled into a 21 item self-administered questionnaire regarding symptoms of abnormal laryngeal sensation.

Item scaling: The items were rated on a 7 point Likert scale where *1* equates to *all of the time* and 7 equates to *none of the time* (Appendix I)*. *A lower score denotes greater impairment with sensory symptoms. This scaling is similar to the Leicester Cough Questionnaire.

Participants completed the 21 item questionnaire prior to their initial assessment in speech pathology. Reproducibility was assessed in a subgroup.

In order to document the changes following therapy a revised 14 item version of the questionnaire was administered to a further group of 44 participants with laryngeal dysfunction syndromes.

### Statistical analysis

Descriptive statistics were obtained for each item. Items with a low mean rating i.e. that indicates the symptom occurs more frequently, were compared to other symptoms to determine whether there was a correlation. A correlation matrix was conducted and rotation component matrix was performed. Factor analysis and item reduction were also completed. Discriminant validity was assessed by one way anova which was conducted on the final questionnaire to determine whether there was a significant difference (1) between the clinical groups, and (2) between the clinical groups and healthy controls. Questionnaire responsiveness was assessed by comparing pre-post treatment data using a Wilcoxon test. Significance was accepted at p < 0.05.

## Results

### Item generation

#### Participant characteristics

Participant characteristics are reported in Table 1. The mean age was 56 years and the majority of participants were female (77%). The dominant symptoms were cough, breathing, voice and globus/swallowing difficulties. The predominant comorbidities were gastroesophageal reflux disease and asthma. It should be noted that these conditions persisted despite medical treatment. Previous medical treatment for the predominant symptom is reported in Table 1.

**Table 1:** Participant characteristics

**Characteristic**	**Group 1: Discriminant analysis**	**Group 2: Responsiveness**
	**N = 38**	**N = 44**
Age M (SD)	56.11 (12.4)	60.4 (13.2)
Gender (% female)	77	73
Dominant Symptom*	Cough 19 (54%)	Cough 38 (86%)
	Breathing 8 (23%)	Breathing 4 (9%)
		Globus/swallow 2 (5%)
	Globus/swallow 5 (14%)	
Smoking	Yes 4 (11%)	No 44 (100%)
	No 28 (80%)	
	Passive 3 (9%)	
Associated medical conditions/previous medical history**	Reflux: 17 (49%); Asthma:10 (29%); Rhinitis: 6 (17%); History of psychiatric disorder: 5 (14%); Obstructive Sleep Apnoea: 4 (11%); unrelated comorbidities: 13 (34%).	Reflux: 9 (21%); Asthma: 12 (27%); Rhinitis: 12 (27%); History of psychiatric disorder: 9 (21%); Bronchiectasis: 1 (2%); unrelated comorbidities: 5 (11)
Other treatment	Asthma treatment (inhaled bronchodilators or inhaled corticosteroids): 17 (49%); Proton pump inhibitor: 19 (54%); Antihistamines: (3%); Nasal/sinus treatment: 5 (14%); Antibiotics: (11%); Continuous Positive Airway Pressure: 1 (3%); Lifestyle changes for reflux: 1 (3%).	Asthma treatment 12 (27%); Proton pump inhibitor 12 (27%); antihistamines 12 (27%)

Note. *Three patients had mixed symptoms. **All associated medical conditions had been treated.

### Item scores

**Figure 1:**
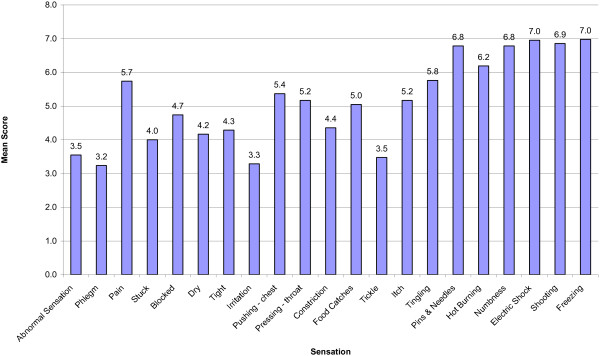
Mean scores for the 21 items for participants in the clinical groups.

The mean scores for each questionnaire item are shown in Table 2. The most common descriptions were *abnormal sensation in the throat*, *phlegm and mucous in the throat*, *tickle and irritation in the throat* and *a tickle in the throat* (Figure 1). The median response for abnormal sensation, phlegm and irritation was 3.0 which indicates that the majority of patients rated that item occurring at least *a good bit of the time.* There was a mixed pattern of responses to *a sensation of something stuck, blocked, dry, tight* and *constriction*, where the frequency of occurrence was relatively equally spread amongst all severity categories. There was infrequent occurrence of *pushing on the chest, pressing on the throat, food catching, itch* and *tingle*. The majority of these participants rated these items between 4.0 and 7.0 ranging from *some of the time* to *none of the time*. These were considered symptoms of neural activation and there were a small number of participants reporting occurrence of the sensation. Finally, there were some items that were typical of pain and thermal neuronal activation that no participants rated as occurring frequently. These items included *pain, pins and needles, hot burning, numbness, shock, shooting*, and *freezing*.

**Table 2:** Descriptive statistics and correlations for the 21 items for participants (excluding healthy controls) in the item generation and discriminant analysis component of the study (n = 38; group 1)

	**Mean**	**SD**	**Correlation with abnormal sensation**	**Correlation with irritation**	**Correlation with phlegm**	**Correlation with tickle**
Abnormal sensation	3.7	1.9		0.6	0.2	0.4
Phlegm and mucous in throat	3.3	1.8	0.2	0.3		0.5
Pain	5.8	1.6	0.3	0.3	0.1	0.3
Sensation of something stuck	4.2	1.9	**0.7**	**0.7**	0.3	0.4
Blocked	4.8	1.9	0.6	0.6	0.1	0.4
Dry	4.3	2.1	0.1	0.2	0.1	0.4
Tight	4.4	1.9	0.6	**0.7**	0.1	0.4
Irritation	3.3	1.6	0.6	1.0	0.3	0.5
Pushing chest	5.4	1.5	0.0	0.3	0.1	0.4
Pressing throat	5.2	1.9	0.5	0.5	0.0	0.1
Constriction	4.4	1.8	0.3	0.3	0.2	0.3
Food catches	5.2	1.6	0.4	0.5	0.2	0.2
Tickle	3.6	1.7	0.4	0.5	0.5	
Itch	5.3	2.0	0.4	0.3	0.5	0.6
Tingling	5.8	1.6	0.4	0.4	0.3	0.5
Pins and needles	6.8	0.7	0.3	0.3	0.3	0.4
Hot burning	6.3	1.2	0.2	0.1	−0.1	0.1
Numbness	6.8	0.5	0.2	0.2	0.3	0.4
Electric shock	6.9	0.3	0.1	0.0	0.2	0.1
Shooting	6.8	0.5	0.1	−0.1	−0.1	0.0
Freezing	7.0	0.2	−0.1	−0.3	−0.2	−0.1

Correlations with the most commonly occurring items are reported in Table 2. *Abnormal sensation* correlated most with ‘a sensation of something stuck in the throat’. Irritation correlated most with ‘a sensation of tightness’.

### Item reduction

Item reduction and factor analysis were then conducted. Of the 21 items, five were excluded due to skewed distribution. These items were *pins and needles, hot burning, numb, shock, shooting* and *freezing*. Thus 16 items were retained for further analysis. An item-item correlation matrix was conducted (Table 3). No items were excluded from this analysis as no items had consistently low correlations. The percentage of items with correlations < 0.2 is reported in Table 3.

**Table 3:** Item-item correlation matrix of the 16 items retained following item reduction and factor analysis in the item generation component of the study

	**Abnsens**	**Phlegm**	**Pain**	**Stuck**	**Blocked**	**Dry**	**Tight**	**Irritation**	**Pushing on chest**	**Pressing on throat**	**Constriction**	**Food catches**	**Tickle**	**Itch**	**Tingle**	**Hot burning**	**No. (%) correlation < .2**
Abnormal sensation	1	0.298	0.307	0.707	0.621	0.114	0.644	0.569	0.087	0.523	0.344	0.399	0.448	0.337	0.414	0.184	3 (19)
Phlegm	0.298	1	0.092	0.299	0.259	0.184	0.187	0.296	0.211	0.080	0.309	0.169	0.491	0.395	0.361	0.035	6 (38)
Pain	0.307	0.092	1	0.304	0.299	0.401	0.405	0.338	0.449	0.414	0.219	0.219	0.317	0.493	0.545	0.637	1 (6)
Stuck	0.707	0.299	0.304	1	0.723	0.010	0.649	0.665	0.130	0.617	0.406	0.457	0.471	0.368	0.484	0.059	3 (19)
Blocked	0.621	0.259	0.299	0.723	1	0.099	0.729	0.617	0.301	0.687	0.614	0.531	0.462	0.304	0.489	0.182	2 (13)
Dry	0.114	0.184	0.401	0.010	0.099	1	0.354	0.230	0.445	0.070	0.149	0.346	0.454	0.404	0.329	0.373	6 (38)
Tight	0.644	0.187	0.405	0.649	0.729	0.354	1	0.714	0.493	0.802	0.588	0.529	0.488	0.461	0.505	0.346	1 (6)
Irritated	0.569	0.296	0.338	0.666	0.617	0.230	0.714	1	0.292	0.529	0.315	0.485	0.517	0.314	0.386	0.168	1 (6)
Pushing On chest	0.087	0.211	0.449	0.130	0.302	0.445	0.493	0.292	1	0.454	0.410	0.114	0.414	0.384	0.313	0.621	3 (19)
Pressing on throat	0.523	0.080	0.414	0.617	0.687	0.070	0.802	0.529	0.454	1	0.664	0.482	0.220	0.413	0.607	0.342	2 (13)
Constriction	0.344	0.309	0.219	0.406	0.614	0.149	0.588	0.315	0.410	0.664	1	0.379	0.323	0.377	0.418	0.218	1 (6)
Food catches	0.399	0.169	0.219	0.457	0.531	0.346	0.529	0.485	0.114	0.482	0.379	1	0.241	0.516	0.485	0.161	3 (19)
Tickle	0.448	0.491	0.317	0.471	0.462	0.454	0.488	0.517	0.414	0.220	0.323	0.241	1	0.542	0.489	0.185	1 (6)
Itch	0.337	0.395	0.493	0.368	0.304	0.404	0.461	0.314	0.384	0.413	0.377	0.516	0.542	1	0.725	0.310	0 (0)
Tingle	0.415	0.361	0.545	0.484	0.489	0.329	0.505	0.386	0.313	0.607	0.418	0.485	0.489	0.725	1	0.364	0 (0)
Hot burning	0.184	0.035	0.637	0.059	0.183	0.373	0.346	0.168	0.621	0.342	0.218	0.161	0.185	0.310	0.364	1	7 (44)

(N = 38, group 1).

Item reliability statistics are presented in Table 4. An item-total correlation was conducted (Table 4), and no items were excluded as all items had correlation coefficients > 0.2. Item reliability was assessed using Cronbach’s alpha, with assessment of reliability with item removal (Table 4). Cronbach’s alpha was high for all items. There was a slight reduction in all items with the exception of *phlegm* and *dry throat*. There was no substantial increase with removal of these items and hence all items were retained.

**Table 4:** Item reliability statistics for the 16 items in the item generation component of the study, (n = 38, group 1)

	**Scale mean if item deleted**	**Scale variance if item deleted**	**Corrected item-total correlation**	**Squared multiple correlation**	**Cronbach's alpha if item deleted**
Abnormal sensation	70.36	300.479	.616	.661	.904
Phlegm	70.62	316.388	.367	.470	.912
Pain	68.07	312.166	.538	.631	.906
Stuck	69.86	297.638	.656	.736	.902
Blocked	69.17	293.654	.717	.769	.900
Dry	69.60	311.808	.378	.650	.913
Tight	69.62	288.046	.824	.870	.896
Irritated	70.60	306.296	.665	.688	.903
Pushing on chest	68.48	312.353	.507	.746	.907
Pressing on throat	68.79	293.343	.708	.898	.900
Constriction	69.57	302.348	.589	.630	.905
Food catches	68.74	308.735	.570	.664	.905
Tickle	70.33	305.886	.624	.731	.904
Itch	68.60	298.442	.644	.742	.903
Tingle	68.19	300.304	.711	.782	.901
Hot burning	67.64	324.333	.413	.637	.909

### Factor analysis

A rotated component matrix was performed (Table 5). Three factors were identified. The items loading on each factor were selected using the highest component. The factors are as follows:

**Table 5:** Rotated component matrix in the item generation component of the study, (n = 38, group 1)

		**Component**	
	**1 Obstruction**	**2 Pain/thermal**	**3 Irritation**
Abnormal sensation	.744		
Pain		.727	
Stuck	.845		
Blocked	.867		
Tight	.812	.372	
Irritated	.710		
Pushing on chest		.773	
Pressing on throat	.836	.384	
Constriction	.621		
Food catches	.576		
Tickle	.328		.752
Itch	.304	.439	.602
Tingle	.480	.425	.446
Hot burning		.856	
Phlegm			.768
Dry		.578	.501

Factor 1: Abnormal sensation, stuck, blocked, tight, irritated, pressing throat, constriction, food catches. These issues were broadly categorised as *obstruction*.

Factor 2: Pain, pushing chest, hot burning. These issues were broadly categorised as *pain/thermal.*

Factor 3: Tickle, itch, phlegm. These factors were broadly classified as *irritation.*

The items *tingle* and *dry* were discarded from the list of 16 items as they loaded evenly across all three factors. The final questionnaire therefore included 14 items loaded across the three factors of *obstruction*, *pain/thermal* and *irritation* (Appendix II).

### Discriminant analysis

**Figure 2:**
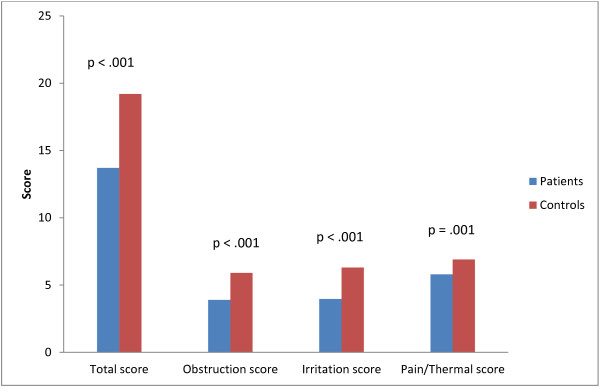
Comparison of total and domain scores for the patient and control groups.

Discriminant validity was assessed by comparing mean scores between each patient group, and healthy controls, for the final 14-item questionnaire (Table 6). These results show that there is no significant difference in any item between clinical groups, however there is a significant difference between healthy controls. Scores were significantly higher (better) in the healthy controls than the clinical groups, indicating discriminant validity of the final questionnaire (Figure 2). The mean (SD) difference between cases and controls was 5.5; (clinical cases 13.7 (3.2): controls 19.2 (0.7), Figure 2.

**Table 6:** Comparison of item, subscale and total scores between participant groups M (SD) in the discriminant analysis component of the study, n = 53

	**Chronic cough**	**PVFM**	**Globus**	**MTD**	**Controls**	**P value**^**1**^	**P value**^**2**^
	**N = 11**	**N = 18**	**N = 6**	**N = 3**	**N = 15**		
Abnormal sensation	4.0 (2.0)	3.6 (2.0)	2.8 (1.5)	2.7 (0.6)	6.7 (0.5)	.366	< .001
Phlegm	2.5 (1.2)	3.9 (2.1)	2.2 (1.3)	4.0 (1.7)	6.1 (1.0)	.146	< .001
Pain	5.6 (1.6)	5.6 (1.8)	6.4 (0.6)	6.0 (1.0)	6.8 (0.4)	.517	.113
Stuck	4.2 (1.8)	4.0 (2.2)	3.6 (1.8)	4.3 (1.5)	6.8 (0.4)	.794	< .001
Blocked	4.9 (2.0)	4.4 (2.1)	5.4 (1.3)	5.0 (1.7)	6.9 (0.3)	.912	.001
Tight	4.9 (2.0)	3.9 (1.8)	4.4 (1.8)	4.3 (2.5)	6.9 (0.4)	.837	< .001
Irritated	3.6 (1.5)	3.1 (1.5)	2.8 (1.5)	4.7 (2.1)	6.3 (0.5)	.280	< .001
Pushing on chest	6.1 (1.3)	5.0 (1.8)	5.8 (1.3)	5.0 (1.7)	7.0 (0.0)	.461	.001
Pressing on throat	6.1 (1.8)	4.6 (2.1)	5.8 (1.1)	5.0 (1.7)	6.9 (0.5)	.586	.002
Constriction	5.0 (1.6)	3.7 (1.9)	6.2 (0.8)	4.3 (1.5)	6.7 (0.7)	.153	< .001
Food catches	4.9 (1.6)	5.1 (1.7)	5.2 (1.8)	5.3 (2.1)	6.7 (0.7)	.857	.018
Tickle	3.7 (2.3)	3.5 (1.5)	3.0 (1.9)	3.7 (0.6)	6.4 (0.6)	.812	< .001
Itch	5.6 (1.9)	5.0 (2.0)	5.0 (2.3)	6.3 (0.6)	6.5 (0.5)	.636	.081
Hot burning	6.6 (0.8)	6.1 (1.5)	6.8 (0.5)	5.3 (1.5)	7.0 (0.0)	.115	.040
**Total cumulative score**^**3**^	**67.8 (17.8)**	**61.2 (17.9)**	**65.4 (12.0)**	**66.0 (11.0)**	**93.5 (3.2)**	**.955**	**< .001**
**Pain/thermal cumulative score**^**3**^	**37.3 (12.0)**	**31.0 (15.4)**	**36.2 (6.9)**	**35.7 (7.1)**	**53.7 (1.9)**	**.891**	**.006**
**Obstruction cumulative score**^**3**^	**33.8 (10.7)**	**29.2 (11.6)**	**33.4 (6.2)**	**31.0 (5.3)**	**47.5 (1.8)**	**.901**	**< .001**
**Irritation cumulative score**^**3**^	**11.3 (4.2)**	**12.3 (5.0)**	**10.2 (4.6 )**	**14.0 (2.0)**	**19.0 (1.6)**	**.296**	**< .001**
**Total score**^**4**^	**14.4 (3.3)**	**13.3 (3.6)**	**13.9 (2.5)**	**14.0 (2.0)**	**19.2 (0.7)**	**.951**	**< .001**
**Pain/thermal score**^**5**^	**6.1 (0.9)**	**5.5 (1.5)**	**6.3 (0.6)**	**5.4 (1.4)**	**6.9 (0.1)**	**.430**	**.006**
**Irritation score**^**5**^	**3.8 (1.4)**	**4.1 (1.7)**	**3.4 (1.5)**	**4.7 (0.7)**	**6.3 (0.6)**	**.458**	**< .001**
**Obstruction score**^**5**^	**4.2 (1.3)**	**3.6 (1.5)**	**4.2 (0.8)**	**3.9 (0.6)**	**6.0 (0.2)**	**.901**	**< .001**

Note. ^1^ = Comparison between clinical groups only; ^2^ = Comparison between clinical groups and healthy controls; ^3^ = total of all scores within domain; ^4^ = Total of all subscale scores; ^5^ = total of all scores in domain divided by the total number of items answered by the participant. Total and subscale scores are in bold.

Note: Higher scores denote better performance.

Concurrent validity was examined by comparing results to the Leicester Cough Questionnaire and the John Hunter Hospital Symptom and Frequency Severity Questionnaire. The Pearson Correlation Coefficient showed a correlation between the Laryngeal Hypersensitivity Questionnaire the Leicester Cough Questionnaire of .673 (p < .001) and the John Hunter Hospital Symptom Frequency and Severity questionnaire of –.791 (p < .001).

### Responsiveness analysis

**Figure 3:**
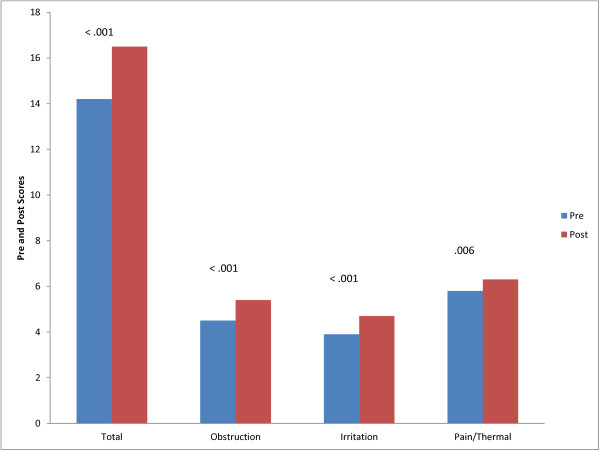
Comparison of pre and post treatment total and domain scores.

Participant characteristics for the responsiveness analysis are reported in Table 1. The Intraclass Correlation Coefficient comparing the two baseline results was .891. Following speech pathology behavioural treatment, there was a significant difference between pre and post Laryngeal Hypersensitivity Questionnaire Scores (p < 0.001; Table 7, Figure 3). The mean pre-post treatment improvement in Newcastle Laryngeal Hypersensitivity Questionnaire scores was 2.3 (SD 3.5; SE .53). Change scores also improved significantly for each factor (Figure 2); obstruction 0.9 (SD 1.3; SE .20), irritation 0.9 (SD 1.5; SE .16), pain/thermal 0.5 (SD 1.1; SE 0.229). The minimally important difference for the total score was calculated at 1.75.

**Table 7:** Comparison of pre-post treatment laryngeal pesthesia questionnaire total scores in the responsiveness component of the study (n = 44 group 2)

**N = 44**	**Pre**	**Post**	**P value**
Mean (SD)	14.1 (3.2)	16.5 (3.3)	< .001 (Repeated t test)
95% CI	13.1 – 15.1	15.4 – 17.5	
Minimum	7.0	8.0	
Maximum	18.0	21.0	< .001 (Wilcoxon)

Note. Higher scores denote better performance. The final 14-item questionnaire contained in Appendix II was used.

## Discussion

The Newcastle Laryngeal Hypersensitivity Questionnaire (LHQ) is a valid measure of laryngeal discomfort for patients with laryngeal hypersensitivity syndromes. The final version contains 14 items with a 7 point Likert frequency response scale. It is designed for self-administration and takes less than 5 minutes for completion. The questionnaire was useful in discriminating between patients with laryngeal hypersensitivity syndromes and healthy controls, and was able to detect a change in laryngeal hypersensitivity after speech pathology treatment.

Healthy controls had a mean total score of 19.2 (SD = 0.7). A cut off for normal function could be considered to be 17.1 (i.e. mean minus 3 standard deviations). Questionnaire scoring is in a similar direction to the Leicester Cough Questionnaire. Subscale scores are averaged from the number of completed items for each subscale and range from 1 (worst) to 7 (best). The total score is the sum of the three subscale scores which range from 3 (worst) to 21 (best). We calculated the minimal important difference as 1.7 using both a standard statistical approach (at 0.5sd), and as the change in LHQ that corresponded to the MID for the Leicester Cough Questionnaire.

The LHQ has a number of potential purposes. It could be used to quantify the patient experience of laryngeal discomfort and to measure change over time. It has the potential to discriminate between patient groups and healthy controls. It could also be used in be used in trials of speech pathology intervention for these clinical groups.

This data also demonstrated that most patients with laryngeal dysfunction have abnormal laryngeal sensation at least *most of the time* although the quality of the sensation is not the same as in chronic pain. It would appear that the laryngeal sensation is similar between clinical groups and may suggest some underlying sensory neuropathy. These findings are consistent with results of quantitative sensory testing in patients with laryngeal hypersensitivity syndrome [7].

Neural hypersensitivity is best characterised in chronic pain syndromes. Symptoms indicating hypersensitivity include a spontaneously occurring sensation, termed pesthesia, increased perception of pain for a given stimulus level, termed hyperalgesia, and a situation where a normally non-painful stimulus evokes pain, termed allodynia. There is growing recognition that laryngeal symptoms in chronic cough can be interpreted in a similar fashion [5,8]. Therefore patients with chronic cough show an increased response to a tussive stimulus such as capsaicin, termed hypertussia, and may also experience cough after exposure to normally nontussive stimuli, such as talking, a symptom termed allotussia. The symptoms rated as important by patients with laryngeal hypersensitivity conform to this pattern. The LHQ contains several examples of laryngeal pesthesia, with symptoms occurring in the absence of a stimulus, such as ‘*an abnormal sensation in my throat’*, ‘*pain in my throat*’, and ‘*my throat feels tight*’. It was beyond the scope of this study to explain causality of symptoms. Although patients had received prior treatment for associated medical conditions such as gastroesophageal reflux disease, asthma, or rhinitis, it is possible that these conditions were contributing to the symptoms. The purpose of the questionnaire was to measure the patient experience of laryngeal symptoms rather than determine the causality of symptoms.

When coupled with a history that these symptoms develop with nontussive triggers (allotussia), or as an exaggerated response to stimuli (hypertussia), the LHQ can aid in the recognition of a laryngeal hypersensitivity syndrome with central sensitisation. This has important treatment implications as recent treatment developments successfully apply the treatment approaches used in chronic pain to chronic refractory cough, such as behavioural therapy [13] or gabapentin [16]. It is also important for understanding mechanisms and new treatment developments [5].

These concepts can be also applied to other laryngeal hypersensitivity syndromes, such as PVFM, muscle tension dysphonia, and globus. We have recently reported evidence for laryngeal pesthesia in these conditions [7] as well as cross stimulus responses which strongly indicate underlying central reflex sensitisation. The results of this study show that the symptoms of laryngeal hypersensitivity as similar in chronic refractory cough, PVFM, muscle tension dysphonia and globus, further supporting the concept of a common underlying laryngeal hypersensitivity in these conditions. It should be noted, however, that the laryngeal hypersensitivity hypothesis is a theory and the heterogeneous patient grouping is only justifiable if the laryngeal hypersensitivity theory is correct. The development of the LHQ will therefore be important in further studying these conditions and assessing responses to treatment.

### Study limitations

One potential limitation of this study is the small sample size for some of the disease groups. This can be overcome by future research that assesses the LHQ in muscle tension dysphonia and globus. The sample size used reflected the referral patterns and community prevalence of these conditions. The patient mix, while heterogeneous, was reflective of overlapping symptomatology and consistent with the multiple underlying medical factors which affect this population. It is possible that this group may not be representative of any particular disorder. Full thematic analysis was not conducted in the item generation phase of the study. Further study of the MID would be useful.

## Conclusion

In conclusion, the study reports the development and validation of a tool to measure laryngeal hypersensitivity that can readily be applied in clinical practice and research. The LHQ will facilitate the recognition and assessment of laryngeal hypersensitivity in several laryngeal disorders and should be useful in as an outcome measure in clinical trials.

## Appendix I

1. There is an abnormal sensation in my throat.

(circle one)

All of the time. 1

Most of the time. 2

A good bit of the time. 3

Some of the time. 4

A little of the time. 5

Hardly any of the time. 6

None of the time. 7

2. I feel phlegm and mucous in my throat

3. I have pain in my throat

4. I have a sensation of something stuck in my throat

5. My throat is blocked.

6. My mouth and/or throat feels dry.

7. My throat feels tight.

8. There is an irritation in my throat.

9. I have a sensation of something pushing on my chest.

10. I have a sensation of something pressing on my throat

11. There is a feeling of constriction as though needing to inhale a large amount of air.

12. Food catches when I eat or drink.

13. There is a tickle in my throat.

14. There is an itch in my throat.

15. I have a tingling sensation in my throat

16. I have pins and needles in my throat

17. I have a hot or burning sensation in my throat.

18. I have numbness in my throat

19. I have a sensation of an electric shock in my throat.

20. There is a shooting sensation in my throat

21. There is a freezing or painfully cold sensation in my throat.

## Appendix II

John Hunter Hospital Laryngeal pesthesia Questionnaire.

1. There is an abnormal sensation in my throat. (O)

(circle one)

All of the time……………………………………………… 1

Most of the time……………………………………………… 2

A good bit of the time……………………………………………… 3

Some of the time……………………………………………… 4

A little of the time……………………………………………… 5

Hardly any of the time……………………………………………… 6

None of the time……………………………………………… 7

2. I feel phlegm and mucous in my throat (TT)

(circle one)

All of the time……………………………………………… 1

Most of the time……………………………………………… 2

A good bit of the time……………………………………………… 3

Some of the time……………………………………………… 4

A little of the time……………………………………………… 5

Hardly any of the time……………………………………………… 6

None of the time……………………………………………… 7

3. I have pain in my throat (P/Th)

(circle one)

All of the time……………………………………………… 1

Most of the time……………………………………………… 2

A good bit of the time……………………………………………… 3

Some of the time……………………………………………… 4

A little of the time……………………………………………… 5

Hardly any of the time……………………………………………… 6

None of the time……………………………………………… 7

4. I have a sensation of something stuck in my throat (O)

(circle one)

All of the time……………………………………………… 1

Most of the time……………………………………………… 2

A good bit of the time……………………………………………… 3

Some of the time……………………………………………… 4

A little of the time……………………………………………… 5

Hardly any of the time……………………………………………… 6

None of the time……………………………………………… 7

5. My throat is blocked. (O)

(circle one)

All of the time……………………………………………… 1

Most of the time……………………………………………… 2

A good bit of the time……………………………………………… 3

Some of the time……………………………………………… 4

A little of the time……………………………………………… 5

Hardly any of the time……………………………………………… 6

None of the time……………………………………………… 7

6. My throat feels tight. (O)

(circle one)

All of the time……………………………………………… 1

Most of the time……………………………………………… 2

A good bit of the time……………………………………………… 3

Some of the time……………………………………………… 4

A little of the time……………………………………………… 5

Hardly any of the time……………………………………………… 6

None of the time……………………………………………… 7

7. There is an irritation in my throat. (O)

(circle one)

All of the time……………………………………………… 1

Most of the time……………………………………………… 2

A good bit of the time……………………………………………… 3

Some of the time……………………………………………… 4

A little of the time……………………………………………… 5

Hardly any of the time……………………………………………… 6

None of the time……………………………………………… 7

8. I have a sensation of something pushing on my chest. (P/Th)

(circle one)

All of the time……………………………………………… 1

Most of the time……………………………………………… 2

A good bit of the time……………………………………………… 3

Some of the time……………………………………………… 4

A little of the time……………………………………………… 5

Hardly any of the time……………………………………………… 6

None of the timee……………………………………………… 7

9. I have a sensation of something pressing on my throat (O)

(circle one)

All of the time……………………………………………… 1

Most of the time……………………………………………… 2

A good bit of the time……………………………………………… 3

Some of the time……………………………………………… 4

A little of the time………………………………………………. 5

Hardly any of the time……………………………………………… 6

None of the time……………………………………………… 7

10. There is a feeling of constriction as though needing to inhale a large amount of air. (O)

(circle one)

All of the time……………………………………………… 1

Most of the time……………………………………………… 2

A good bit of the time……………………………………………… 3

Some of the time……………………………………………… 4

A little of the time……………………………………………… 5

Hardly any of the time……………………………………………… 6

None of the time……………………………………………… 7

11. Food catches when I eat or drink. (O)

(circle one)

All of the time……………………………………………… 1

Most of the time……………………………………………… 2

A good bit of the time……………………………………………… 3

Some of the time……………………………………………… 4

A little of the time……………………………………………… 5

Hardly any of the time……………………………………………… 6

None of the time……………………………………………… 7

12. There is a tickle in my throat. (TT)

(circle one)

All of the time……………………………………………… 1

Most of the time……………………………………………… 2

A good bit of the time……………………………………………… 3

Some of the time……………………………………………… 4

A little of the time……………………………………………… 5

Hardly any of the time……………………………………………… 6

None of the time……………………………………………… 7

13. There is an itch in my throat. (TT)

(circle one)

All of the time……………………………………………… 1

Most of the time……………………………………………… 2

A good bit of the time……………………………………………… 3

Some of the time……………………………………………… 4

A little of the time……………………………………………… 5

Hardly any of the time……………………………………………… 6

None of the time……………………………………………… 7

14. I have a hot or burning sensation in my throat (P/Th)

(circle one)

All of the time……………………………………………… 1

Most of the time……………………………………………… 2

A good bit of the time……………………………………………… 3

Some of the time……………………………………………… 4

A little of the time……………………………………………… 5

Hardly any of the time……………………………………………… 6

None of the time……………………………………………… 7

## Abbreviations

LHQ: Laryngeal hypersensitivity questionnaire; PVFM: Paradoxical vocal fold movement.

## Competing interests

The authors declare that they have no competing interests.

## Authors’ contributions

AV and PG conceived the study and wrote the manuscript. AV did the statistical analysis. SB was responsible for collection and collation of the data. All authors read and approved the final manuscript.
